# Turning major revision into opportunity: 10 tips to navigate peer review after manuscript submission

**DOI:** 10.1093/ckj/sfag066

**Published:** 2026-02-26

**Authors:** Mythri Shankar, Mohamed Hassanein, Alexander Woywodt

**Affiliations:** Institute of Nephro Urology, Department of Nephrology, Bengaluru, KA, India; Kidney and Urology Center, Department of Nephrology and Hypertension, Alexandria, Egypt; Royal Preston Hospital, Department of Renal Medicine, Royal Preston Hospital, Preston, Lancashire Teaching Hospitals NHS Foundation Trust, UK

To the Editor,

Peer review remains the backbone of scientific publishing, yet authors often face difficult reviews marked by excessive demands, perceived bias or misinterpretation of their work. Such experiences can feel discouraging, particularly when accompanied by a verdict of ‘major revision’, often interpreted as a near–rejection rather than an opportunity for improvement. Viewing peer review as both quality control and constructive dialogue can transform these moments into catalysts for advancing the manuscript’s quality. Major revision is an opportunity, not a defeat. Here, we summarize 10 tips to navigate challenging peer review [1, 2].

First, respect the journal’s decision, whether it results in rejection or revision. Emotional regulation is critical, as negative reviews may trigger anger or defensiveness, especially when the work is closely linked to the author’s identity or career trajectory. A short ‘cooling off’ period helps authors regain objectivity and engage productively with the reviewers’ intellectual content [3].

Once emotions settle, identify the core ‘major’ comments—those crucial to the editorial decision. Distinguish between scientific issues (methods, statistics, interpretation) and communication issues (unclear writing, missing context, inadequate literature positioning). Often, what appears to be a fundamental objection reflects a communication gap repairable through clearer explanation, improved figures or a transparent rationale [4, 5].

Responding with professionalism is essential, even when reviewers seem mistaken or unfair. Standard practice includes a concise, positive cover letter and a numbered, point–by–point response to all comments. The cover letter should restate the study’s unique contribution or clinical relevance, reminding editors why the manuscript matters. Begin by thanking the reviewers and editors for their time and constructive feedback. Quoting each comment verbatim and detailing the corresponding change—supported by a highlighted or tracked version—facilitates re–review and increases the likelihood of acceptance. When authors disagree with a suggestion, they should reply respectfully, provide a clear scientific rationale and propose balanced alternatives. If reviewers’ demands are overwhelming, the editor’s instructions should guide prioritization, and authors should transparently justify their approach in both the manuscript and response document [4].

More challenging are comments that appear unreasonable or biased, such as calls for an entirely new study, critiques beyond scope or personal remarks. Authors can acknowledge the concern, clarify practical or ethical constraints, and, where appropriate, request editorial adjudication. This preserves professionalism while signalling that certain requests exceed what is scientifically warranted. When comments are contradictory or non–scientific, seeking advice from an experienced academic or involving co–authors in reviewing the responses ensures balanced, collegial replies. Substantial revision guided by these principles can turn even harsh reviews into blueprints for a stronger paper [4].

**Figure 1:**
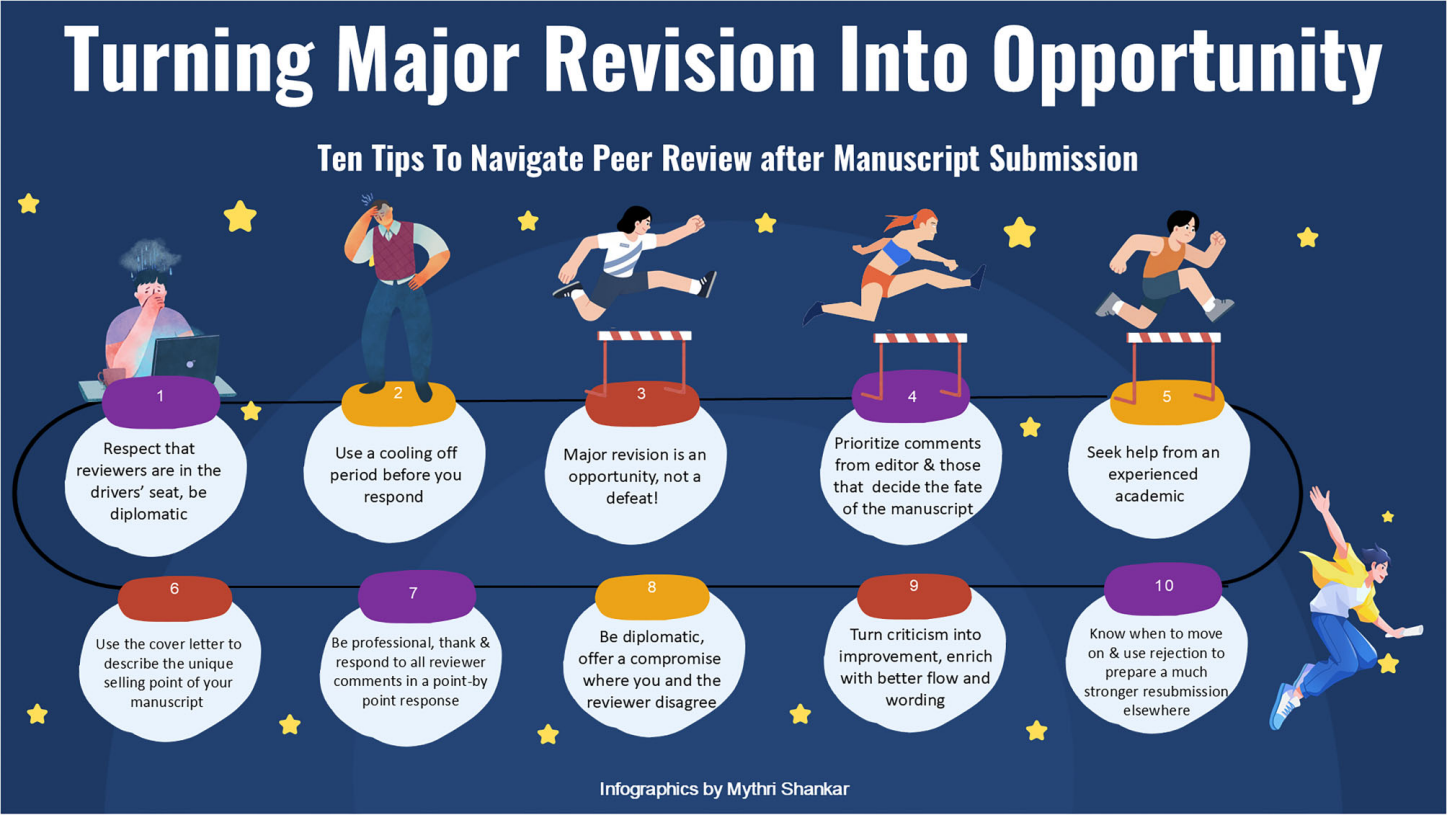
Ten tips to navigate challenging peer review. Infographics by Mythri Shankar.

In Fig. 1, we summarize 10 practical tips to handle tough peer review, from emotion regulation to academic resilience. Common strategies include restructuring sections for better flow, clarifying methods and statistical approaches, strengthening discussion of limitations, and adding robustness or sensitivity analyses when appropriate. A concise, editor–focused cover note that highlights the most significant changes and directly links them to major concerns can further streamline evaluation.

Finally, rejection and repeated revision should be normalized as inherent parts of academic life rather than indicators of failure. When a manuscript is ultimately rejected, the revised version—shaped by reviewers’ critiques—often becomes a stronger submission to a more suitable journal. Constructive engagement with peer review enhances not only a single paper’s quality but also long–term skills in writing, critical thinking and resilience. For clinical researchers and nephrologists, adopting this mindset enables us to navigate challenging reviews with confidence, strengthen the scientific foundation of our work and contribute meaningfully to advancing global kidney care [4, 5].
